# Glucose affects capsular polysaccharides synthesis via CcpA and HPr in *Streptococcus pneumoniae*

**DOI:** 10.71150/jm.2411024

**Published:** 2025-05-27

**Authors:** Rui Yang, Yapeng Zhang, Hong Wang, Hanyi Wang, Jiangming Xiao, Lian Li, Yuan Yuan, Yibing Yin, Xuemei Zhang

**Affiliations:** 1 Department of Laboratory Medicine, Key Laboratory of Diagnostic Medicine (Ministry of Education), Chongqing Medical University, Chongqing 404100, P. R. China; 2 Department of Clinical Laboratory, Affiliated Hospital 6 of Nantong University, Yancheng Third People’s Hospital, Yancheng 224000, P. R. China; 3 Department of Clinical Laboratory, The First People's Hospital of Chongqing Liang Jiang New Area, Chongqing 404100, P. R. China; 4 Department of Clinical Laboratory, The Seventh People's Hospital of Chongqing, Chongqing 404100, P. R. China

**Keywords:** *Streptococcus pneumoniae*, capsular polysaccharide, CcpA, glucose, HPr

## Abstract

*Streptococcus pneumoniae* is a conditionally pathogenic bacteria that colonizes the nasopharynx of 27% to 65% of children and 10% of adults. Capsular polysaccharides are the most critical virulence factor of *S. pneumoniae*, and nonencapsulated strains are usually non-pathogenic. Previous studies have shown that glucose regulates capsule synthesis. To investigate the mechanism of carbon metabolism regulatory factors CcpA and HPr regulating capsule synthesis in the presence of glucose as the sole carbon source, we constructed deletion mutants (D39Δ*ccpA* and Δ*ptsH*) and complemented strains (D39Δ*ccpA::ccpA* and Δ*ptsH::ptsH*). In this study, we found that the promoting effect of capsule synthesis by glucose disappeared after the deletion of *ccpA* and *ptsH*, and demonstrated that the protein CcpA regulates capsule synthesis by binding to the *cps* promoter and altering the transcription level of the *cps* gene cluster. Increased glucose concentration up-regulated the level of HPr-Ser46~P, which enhanced the binding ability of CcpA to the DNA sequence of the *cps* promoter, thus promoting capsule synthesis. HPr also has a regulatory effect on capsule synthesis. These insights reveal a new synthesis mechanism of capsular polysaccharide and provide a new strategy of antibacterial drugs for *S. pneumoniae*.

## Introduction

*Streptococcus pneumoniae* is a Gram-positive bacteria that colonizes the nasopharynx extensively and is transmitted primarily by inhalation of infectious aerosols or direct contact with infected mucous membranes or secretions. It can invade and infect multiple organs, including the middle ear, lungs, bloodstream, heart, and brain, often resulting in mucosal infections and invasive infections in both children and adults (Dao and Rosch, 2021). *S. pneumoniae* colonization and infection are endemic to all regions and are the leading cause of pneumonia deaths worldwide (Troeger et al., 2018), with more than 1.5 million fatalities each year attributed to invasive and fatal pneumococcal disease (Butters et al., 2019; Wahl et al., 2018). Traditional treatments for *S. pneumoniae* infection include antibiotic therapy and vaccines. However, current studies have shown that *S. pneumoniae* has developed resistance to a variety of antibiotics, including β-lactams, macrolides, and tetracyclines (Zhou et al., 2022). To address the diversity of capsular polysaccharides and prevent infections caused by multiple serotypes, the development of vaccines is crucial. Unfortunately, commercially available vaccines still have limitations, such as restricted serotype coverage and serotype substitution (Weinberger et al., 2011), so there is an urgent need to find new targets for the development of antimicrobial drugs.

Capsular polysaccharides (CPS) is the major virulence factor required for effective colonization and induction of invasive disease (de Vos et al., 2015; Hyams et al., 2010; Wartha et al., 2007). Nonencapsulated strains are usually non-pathogenic. Therefore, to explore the synthetic regulatory mechanism helps to find new antibacterial targets.

Current studies have identified 109 serotypes in *S. pneumoniae* (Su et al., 2021). Most CPS is synthesized via the Wzx/Wzy-dependent pathway, and the genes involved in CPS synthesis are located between *dexB* and *aliA*, except for serotype 37 (Geno et al., 2015; Yother, 2011). *cps* gene cluster is involved in the formation of CPS and its expression is driven by a single *cps* promoter (Shainheit et al., 2014). It has been reported that the transcript levels of *cps* genes can be affected by deletion of individual elements between the coding sequences of *dexB* and *cps2A* (Wen et al., 2015). Bioinformatics analysis showed that there were motifs similar to transcription regulatory binding sites in the promoter region of *cps* gene cluster, such as RitR, MalR, CopY, and AdcR, which need to be confirmed by relevant experimental evidence (Moscoso and Garcia, 2009). CpsR and phosphorylated ComE have been confirmed to bind specifically to the *cps* promoter sequence and negatively regulate CPS synthesis (Wu et al., 2016; Zheng et al., 2017).

Several environmental factors, such as oxygen availability (Geno et al., 2014) and carbon sources (Troxler et al., 2019), have been associated to regulate *cps* gene transcription. CcpA (Catabolite Control Protein A) is a key regulator of carbon metabolite repression. With the help of the phosphoenolpyruvate-dependent phosphotransferase system (PTS), it binds to the specific DNA sequence to promote or repress the expression of the target gene, thereby regulating the physiological process and virulence of bacteria (Warner and Lolkema, 2003; Yang et al., 2017). Previous studies have confirmed that CcpA has a significant impact on the capsule synthesis and virulence in *Streptococcus suis*, with the absence of its activity leading to reduced capsule expression and decreased resistance to phagocytosis and killing (Willenborg et al., 2011). In *S. pneumoniae*, the RegM mutation, an analog of CcpA, resulted in a significant reduction in the transcription of *cps* gene cluster and a decrease in virulence (Giammarinaro and Paton, 2002). Our previous studies have demonstrated that CcpA not only affects the natural transformation by regulating the transcription of *comCDE*, but also promotes CPS synthesis in *S. pneumoniae* (Zhang et al., 2023). Therefore, we proposed to explore whether CcpA can sense the sugar signal in *S. pneumoniae* and regulate CPS synthesis accordingly.

HPr is a component of PTS encoded by the *ptsH* gene. During sugar uptake, serine residues of HPr protein are phosphorylated by HPr kinase/phosphatase (HprK/P) to form HPr-Ser46~P and produce a complex with CcpA, which is involved in CCR effects (Chauvaux, 1996). Research has shown that HPr-Ser46~P is not only a central regulator of carbon metabolism, but also plays an significant role in the virulence development of Gram-positive bacteria such as *Streptococcus pyogenes*, *Bacillus subtilis*, and *Listeria monocytogenes* (Deutscher et al., 2005). However, to data, no study has reported whether HPr-Ser46~P affects the regulatory effects of CcpA on CPS synthesis in *S. pneumoniae*.

In this study, we describe the specific ways in which CcpA regulates CPS synthesis in an environment where glucose is the sole carbon source, and explore how *S. pneumoniae* modulates CPS synthesis through CcpA in response to different concentrations of glucose. This regulatory mechanism that requires the presence of the auxiliary protein HPr, which is altered by activation or inhibition of environmental signals, resulting in changes to its phosphorylation levels. These changes subsequently affect the activity of CcpA binding to the *cps* promoter, ultimately influencing CPS synthesis. This study elucidates the molecular mechanism of glucose-regulated CPS synthesis through CcpA and HPr, and provides a new target and rationale for the development of new therapeutic approaches for *S. pneumoniae* disease.

## Materials and Methods

### Bacterial strains and growth conditions

The bacterial strains and plasmids used in this study are listed in Table S1. All *S. pneumoniae* strains were grown at 37°C under 5% CO_2_ in semi-synthetic casein hydrolyzate medium supplemented with 5% yeast extract (C + Y, pH 7.0) medium or plated on blood agar plates. *Escherichia coli* strains were grown in lysogeny broth (LB) with shaking or LB agar plates at 37°C. Selective antibiotics were added when necessary.

### Bacterial strains constructions

The primers used in this study are listed in Table S2. We constructed mutants by generating unlabeled mutations in the pneumococcal genome using the Janus technique. All mutants are derived from the D39s strain, a streptomycin-resistant derivative of D39Δ*ptsH* was produced in a two-step transformation process. First the upstream and downstream sequences of the *ptsH* locus were amplified with primer pairs Pr1440/1441 and Pr1442/1443 from D39s. The Janus cassette was amplified with primers Pr1332 and Pr1333 from genomic DNA of strain ST588. These amplicons were ligated to obtain a recombinant fragment that was transformed into D39s to construct Δ*ptsH*::kana-*rpsL* (Δ*ptsH::JC*). Unmarked deletions in the *ptsH* locus were then constructed by overlapping upstream (primers Pr1440/YR0013) and downstream (primers Pr1443/YR0014) sequences with primers Pr1440/1443 and transformed into strain Δ*ptsH::JC* to construct unmarked deletion strain (Δ*ptsH*).

The *ptsH* complemented strain was constructed by using shuttle plasmid pIB166. DNA fragments containing the *ptsH* gene and its predicted upstream promoter are amplified by PCR with primer YR0019/YR0020. the obtained DNA fragments were digested with suitable restriction enzymes (BamHI and XhoI) and cloned into the shuttle plasmid pIB166 to generate the recombinant. The recombinant plasmids pIB166-*ptsH* was transformed into the Δ*ptsH* mutant strain to construct the *ptsH* complemented strain (Δ*ptsH::ptsH*).

### Recombinant protein production

The pneumococcal *ptsH* gene from *S. pneumoniae* D39 was amplified by PCR and cloned into the pET-28a expression vector and into *E. coli* BL21 (DE3). Expression was induced at an OD_600_ of 0.8 by addition of 1 mM isopropyl-D-thiogalactoside (IPTG) at 20°C by shaking at 120 rpm for 8–10 h. The HPr protein was collected and purified by affinity chromatography with an Ni21/nitrilotriacetic acid (NTA) column. To obtain HPr-Ser46~P protein, we mutated the 136th serine residue (TCA) to aspartic acid residue (GAC) in the *ptsH* gene. The point mutation fragment was confirmed by sequencing and cloned into the pET-28a expression vector and into *E. coli* BL21 (DE3). Subsequent steps were produced in the same way.

### Uronic acid assay

Bacterial strains were cultured to OD_600_ = 0.5 at 37°C and 5% carbon dioxide in C + Y medium to collect 3ml and centrifuged at 4°C and 12,000 rpm for 5 min, removing the supernatant and leaving the pellet. Bacterial precipitations were resuspended in 500 μl of Tris-HCl/MgSO_4_ buffer (containing 150 mM Tris·HCl and 1 mM MgSO_4_, pH 7.0). The samples were added 2 μl sulfamate and 1 ml of borate solution, heated at 100℃ for 15 min, cooled to room temperature, and then added 16 μl m-hydroxybiphenyl solution (0.15%), placed for 30 min, the amount of capsules was detected by measuring absorbance at 525 nm.

### Western blot

Bacterial strains were grown in C + Y medium at 37°C to an OD_600_ of 0.5. The samples were centrifuged, and the cell pellets were lysed with lysis buffer (0.5% deoxycholate). The protein concentration was measured using the NanoDrop spectrophotometer, and the samples were subjected to SDS-PAGE and electrotransferred onto 0.2 mm polyvinylidene difluoride (PVDF) membranes (Merck Millipore, USA). To detect HPr, purified HPr was used to immunize kunming mice to generate polyclonal antibodies that was used diluted at 1:5000. To detect capsule, Pneumococcus Type 2 serum was used diluted at 1:5000. The proteins were visualized by adding Immobilon Western horseradish peroxidase (HRP) substrate peroxide solution (Millipore) captured using Image Lab software (Bio-Rad).

### Quantitative real-time PCR

According to the operating manual, RNA was extracted using the RNAprep Pure Cell/Bacteria Kit (Tiangen). Reverse transcription reactions were performed using the prime script RT Master Mix kit (TaKaRa-Bio). Quantitative PCR reactions were performed using the BioRad CFX Connect PCR instrument, and the primers were quantified as shown in Table S2. Relative changes in gene expression were calculated by the 2^-ΔΔCt^ method using gyrB as an internal reference gene. The results of representative experiments are presented as the means of three replicates ± Standard Error of the Mean.

### Electrophoretic mobility shift assay

The 5′-biotin-labeled and unlabeled DNA fragments containing the promoter region of the *Pcps* probes was amplified from strain D39. Chemiluminescence EMSA kits were purchased from Beyotime. EMSAs are carried out according to the manufacturer’s instructions. The EMSA reaction was performed in a total volume of 10 μl, followed by the addition of ddH_2_O, 5X binding buffer, different concentrations of protein and unlabeled probes. The reaction mixture was incubated for 10 min at room temperature. After incubation, labeled probe was added and the reaction mixture was incubated for 20 min at room temperature. Subsequently, 10 μl of the solution was loaded onto a precast 6% acrylamide gel (Invitrogen) and electrophoresis at 100 V for 60 min. DNA was transferred to a nylon membrane and crosslinked by exposure to ultraviolet (UV) light, and the bands were visualized with a chemiluminescence substrate and captured using the Chemiluminescence imaging system (Bio-Rad).

### Luciferase activity assay

Luciferase reporter construct of the *cps* promoter region was from Tsinghua University. The plasmid pTH3937 was transferred to D39s, Δ*ccpA* and Δ*ptsH* to generate D39s-*Pcps-luc*, D39sΔ*ccpA*-*Pcps-luc* and D39sΔ*ptsH*-*Pcps-luc*. The relative luminescence unit (RLU) values were determined in a GloMax luminescence detector (Promega) by adding 1 μl D-fluorescein (0.66 mM) (Beyotime) to a 100 μl bacterial medium. The optical density (OD_600_) of the samples was measured and used to normalize the luciferase activity.

### Animal experiments

Female C57BL/6 mice (6–8 weeks old, weighing 20–22 g) were purchased from the Laboratory Animal Center of Chongqing Medical University. The Ethics Committee of Chongqing Medical University approved this study.

D39s, Δ*ptsH* and Δ*ptsH::ptsH* strains were grown to 0.5 in C + Y, bacteria were collected by centrifugation, washed and suspended in sterile PBS. For the lung infection model, C57BL/6 mice (6 to 8 weeks old, female) were infected intratracheally with 2 × 10^7^ CFU. For pneumonia model and survival assay, C57BL/6 mice (6 to 8 weeks old, female) were infected intratracheally with 1 × 10^8^ CFU, mouse survival was monitored daily for 14 days. To determine the organ involvement, blood aliquots and nasal lavage samples were collected from mice following induction of general euthanasia at 48 h after infection. Lung and spleen whole tissues were homogenized. Bacterial counts in the blood as well as organ homogenates were determined by separately plating serial dilutions. The results were presented as the means of three replicates ± the standard deviation (SD).

### Statistical analysis

The survival was analyzed using the log-rank test. All others were analyzed using two-tailed unpaired Student’s test, with statistical significance defined as P < 0.05 (*), < 0.01 (**) and < 0.001 (***).

## Results

### CcpA promotes CPS synthesis in an environment with glucose as the sole carbon source

CcpA is a central regulator of the carbon catabolite repression (CCR) and previous studies have shown that CcpA promotes CPS synthesis (Zhang et al., 2023), so we proposed to investigate whether CcpA is able to sense sugar signals to regulate CPS synthesis. Since glucose is the preferred carbon source for most microorganisms, we mainly explored the molecular mechanism by which single glucose regulates CPS via CcpA.

**Fig. 1.**
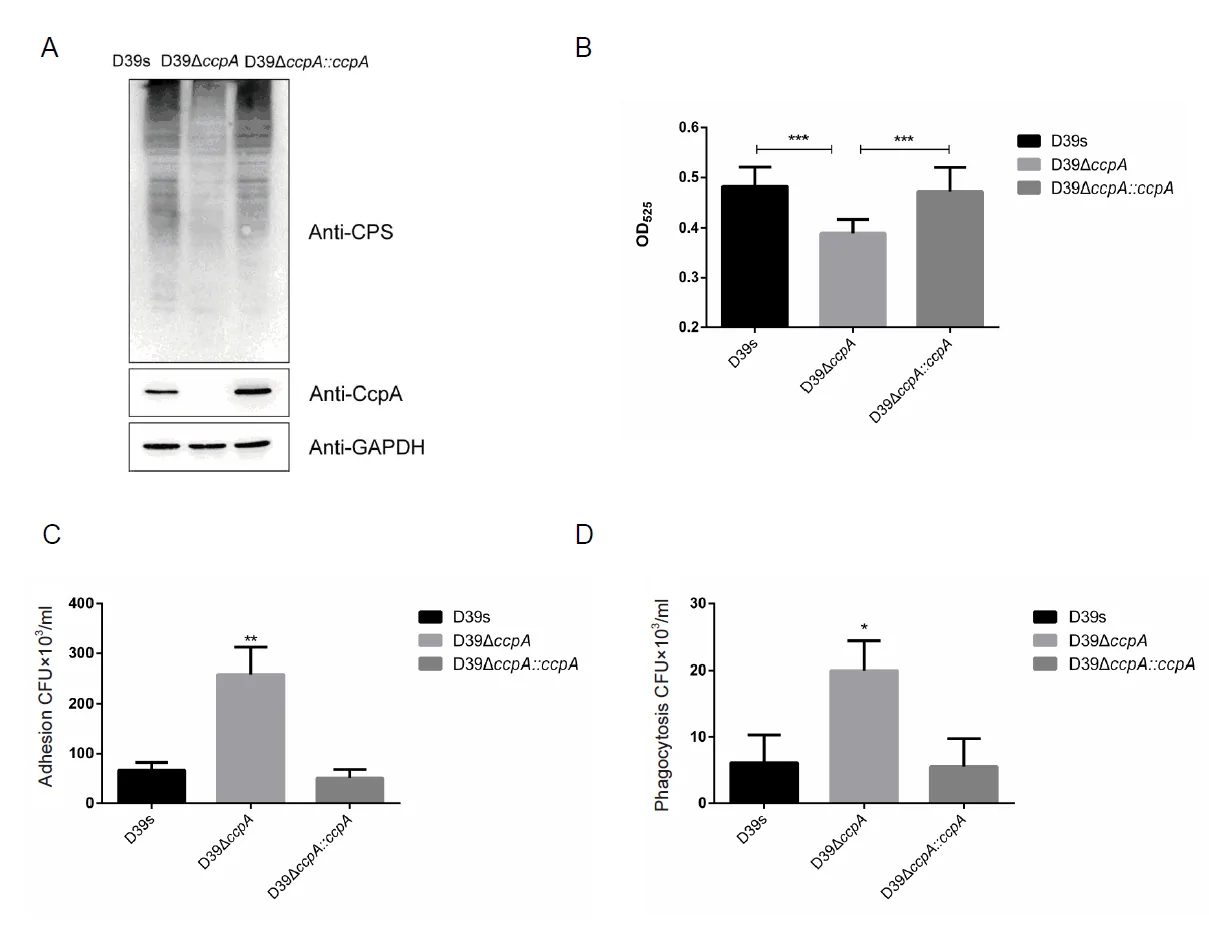
CcpA promotes CPS synthesis in an environment with glucose as the sole carbon source. (A) Western blot to determine the CPS content of D39s, D39Δ*ccpA* and D39Δ*ccpA::ccpA*. (B) Uronic acid assay to determine the CPS content of D39s, D39Δ*ccpA* and D39Δ*ccpA::ccpA*. (C) Macrophage adhesion capacity of D39s, D39Δ*ccpA* and D39Δ*ccpA::ccpA*. (D) Macrophage antiphagocytic capacity of D39s, D39Δ*ccpA* and D39Δ*ccpA::ccpA*. Data are shown as mean ± SD of three independent experiments (n = 3). ^*^, p < 0.05; ^**^, p < 0.01; ^***^, p < 0.001; ns, not significant.

To clarify the effect of CcpA on CPS synthesis in an environment with glucose as the sole carbon source, Western blot was performed to determine the production of CPS in D39s, Δ*ccpA*, and Δ*ccpA::ccpA* in C + Y medium with 10mM glucose as the sole carbon source. The result revealed that the production of CPS in Δ*ccpA* was significantly reduced compared to the wild-type (Fig. 1A). Uronic acid assay also showed a consistent trend (Fig. 1B). These results indicated that CcpA has a promoting effect on CPS synthesis in an environment with glucose as the sole carbon source.

Since CPS is the most important virulence factor of *S. pneumoniae* (Su et al., 2021), inhibition or interference with CPS synthesis significantly attenuates the virulence of *S. pneumoniae*, we assessed the adhesion and antiphagocytic ability of D39s, Δ*ccpA*, and Δ*ccpA::ccpA*. There was a significant increase in the amount of *ccpA*-deficient strain adhering to the macrophage surface or being engulfed into the macrophage. The result coincided with the changes in CPS content, corroborating the positive regulation of CPS synthesis by CcpA (Fig. 1C and 1D).

### CcpA binds to the *cps* promoter to promote transcription of the *cps* operon

**Fig. 2.**
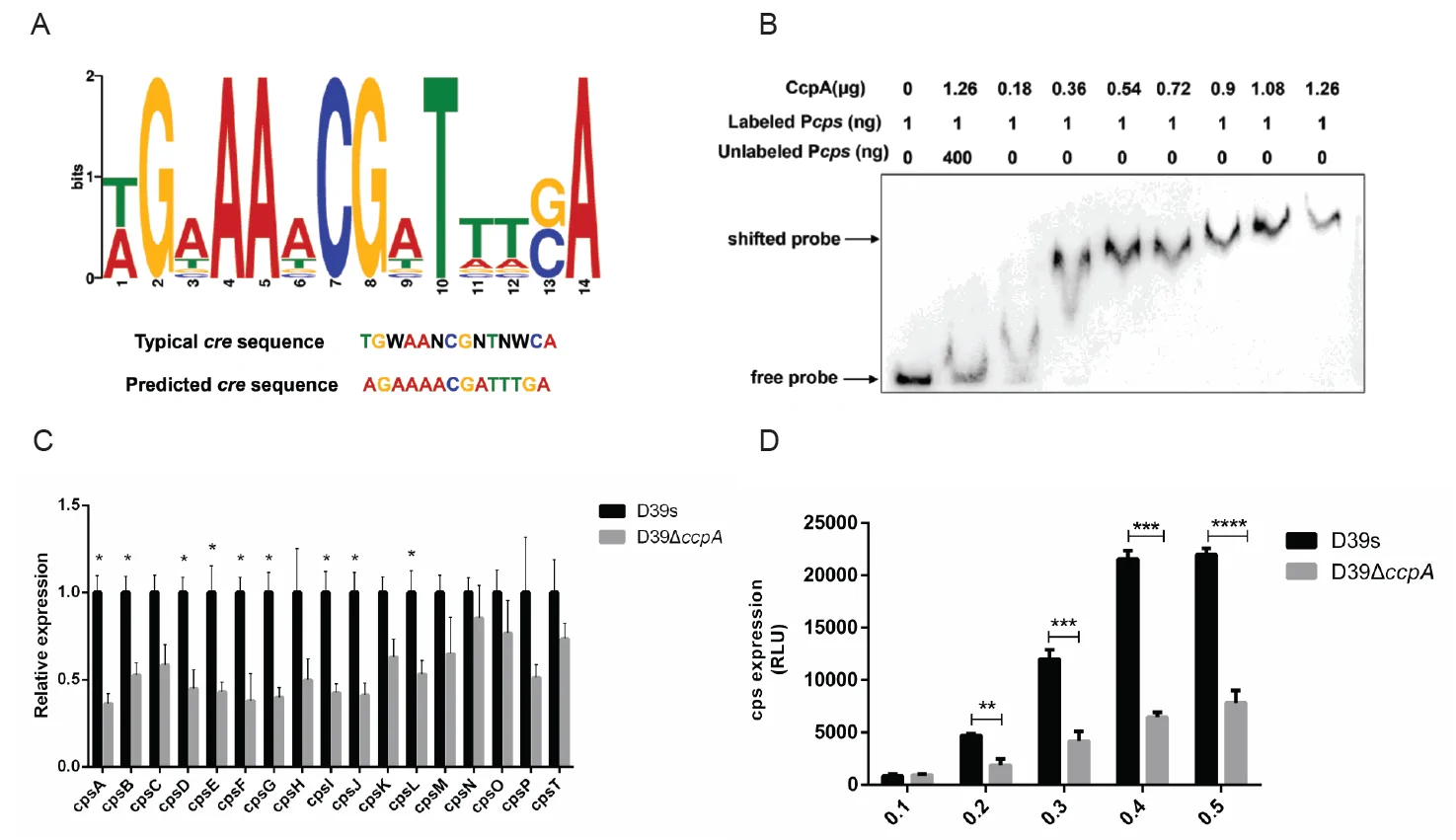
CcpA binds specifically to the *cps* promoter to promote *cps* operon transcription. (A) Typical CcpA binding sites were compared to *cps* promoter sequences using the MEME online site. Typical *cre* sequences and predicted *cre* sequences on the *cps* promoter are shown in the figure, with N representing any base and W representing A or T. (B) EMSA verified the specific binding of CcpA to *cps* promoter. (C) Real-time fluorescence quantitative PCR to detect the transcript levels of genes related to CPS synthesis. (D) Luciferase reporter strains D39s-*Pcps-luc* and Δ*ccpA*-*Pcps-luc* were assayed for *cps* promoter activity. Data are shown as mean ± SD of three independent experiments (n = 3). ^*^, p < 0.05; ^**^, p < 0.01; ^***^, p < 0.001; ns, not significant.

CcpA is a transcriptional regulator that belongs to the LacI/GalR family (Henkin, 1996). Studies have shown that CcpA affects gene expression by binding to cis-regulated DNA known as catabolite responsive elements (*cre*), which consist of a typical consensus site, TGWAANCGNTNWCA (Weickert and Chambliss, 1990). We identified a conserved CcpA-binding sequence (Fig. 2A) in the *cps* promoter region. To further determine whether CcpA specifically binds to the *cps* promoter, we expressed and purified CcpA protein (Fig. S1A), and detected binding ability by gel mobility shift assay (EMSA). As CcpA concentration increased, a significant increase in the DNA-protein complex and the binding of CcpA to the labeled probe was inhibited by adding a non-biotin-labeled competing probe (Fig. 2B). The result indicated the specific binding of CcpA to the *cps* promoter.

Q-PCR was performed to test the expression of several genes in the *cps* locus. The results showed that the expression of genes in *ccpA*-deficient bacteria were all down-regulated compared to the wild-type (Fig. 2C). We further assessed the effect of CcpA on *cps* operon transcription by investigating the activity of *cps* promoter in luciferase reporter strains. Consistent with the Q-PCR results, the luciferase activity of D39Δ*ccpA*-*Pcps-luc* at different growth stages was lower than that of D39s-*Pcps-luc* significantly (Fig. 2D). These results suggested that CcpA may regulate CPS synthesis by specifically binding to the *cps* promoter and promoting the expression of *cps* operons.

### CcpA is involved in the regulation of glucose on capsule synthesis

**Fig. 3.**
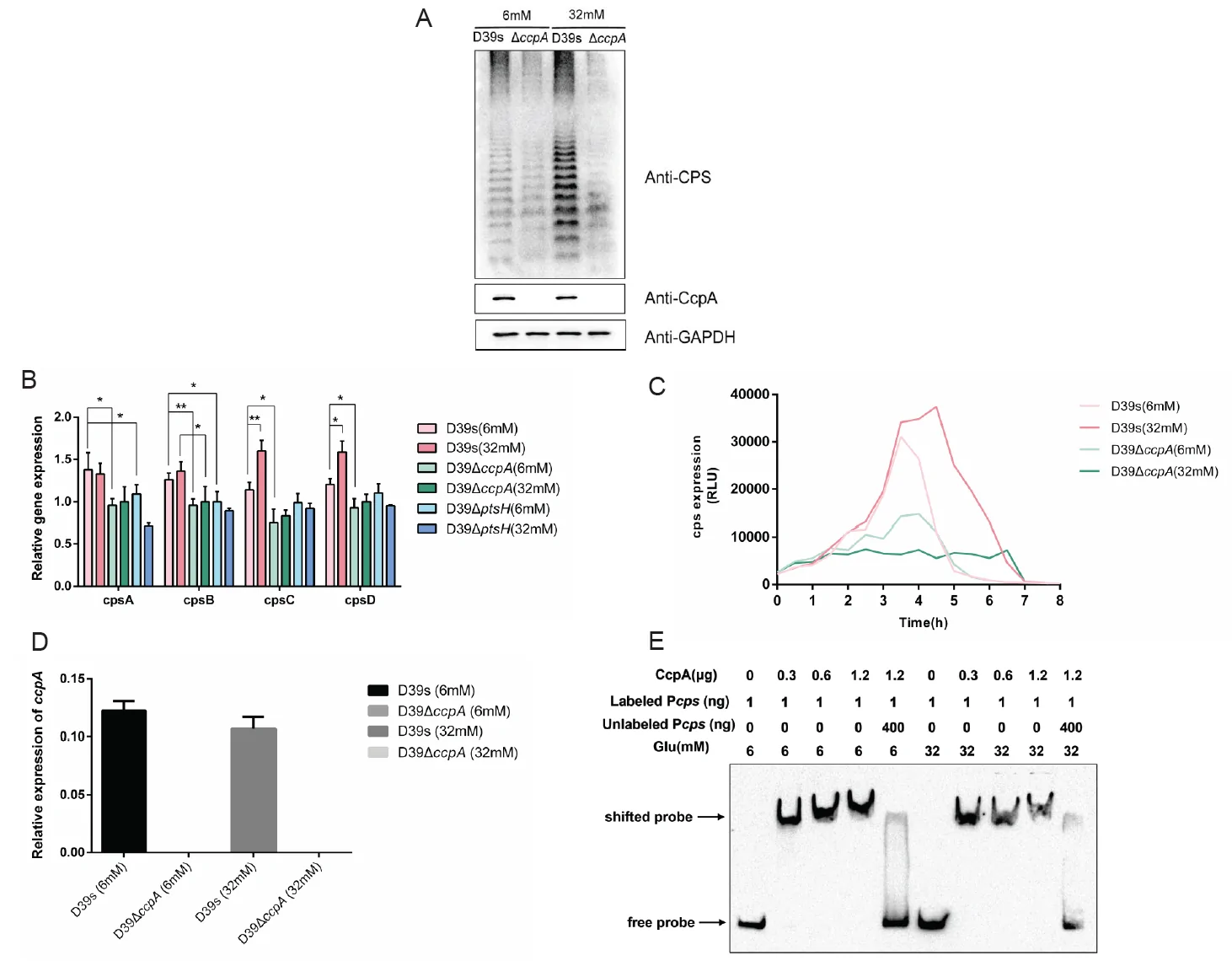
CcpA is involved in the regulation of glucose on capsule synthesis. (A) Western blot assay to determine the CPS expression of D39s and D39Δ*ccpA* at different glucose concentrations. (B) Real-time fluorescence quantitative PCR to detect the transcript levels of CPS synthesis-related genes in D39s, D39Δ*ccpA* and D39Δ*ptsH* at different glucose concentrations. (C) Luciferase reporter strains D39s-*Pcps-luc* and Δ*ccpA*-*Pcps-luc* were assayed for *cps* promoter activity at different glucose concentrations. (D) Real-time fluorescence quantitative PCR to detect the transcript levels of *ccpA* in D39s and D39Δ*ccpA* at different glucose concentrations. (E) EMSA detected the binding of CcpA to *cps* promoter at different glucose concentrations. Data are shown as mean ± SEM of three independent experiments (n = 3). ^*^, p < 0.05; ^**^, p < 0.01; ^***^, p < 0.001; ns, not significant.

To investigate whether glucose regulates the transcriptional levels of genes in the *cps* locus by CcpA, we examined the CPS contents and the mRNA expression of the first four genes of the *cps* operon (cps2A–2D) in wild-type and *ccpA*-deficient strains at 6 mM (low concentration) and 32 mM (high concentration) glucose concentrations, respectively. The result showed that differences in CPS content at different glucose concentrations diminished upon CcpA deficiency (Fig. 3A), and steady-state mRNA levels in wild-type strains for cps2C/D was increased in high glucose environment compared to low glucose environment. In the *ccpA*-deficient strains, there was no significant difference at different glucose concentrations, and mRNA levels were down-regulated in *ccpA*-deficient strains compared with wild-type at a low glucose concentration (Fig. 3B). We further assessed the activity of *cps* promoter. The result indicated that the *cps* promoter activity of the wild-type increased with increasing glucose concentration, whereas was unchanged in Δ*ccpA* mutant. Furthermore, *cps* promoter activities were significantly lower in *ccpA*-deficient strains compared to wild-type (Fig. 3C). In conclusion, increased glucose concentration promoted *cps* operons transcription, and this regulation required the involvement of CcpA.

Therefore, we further explored how glucose affect CcpA-mediated regulation. However, the mRNA expression of *ccpA* remained unchanged (Fig. 3D) and its protein levels had no difference at different glucose concentrations (Fig. 3A). In addition, we found that variations in glucose concentration did not affect the binding of CcpA to the *cps* promoter (Fig. 3E). These results suggested that glucose regulated the effect of CcpA on CPS synthesis in other ways.

### HPr promotes capsule synthesis

**Fig. 4.**
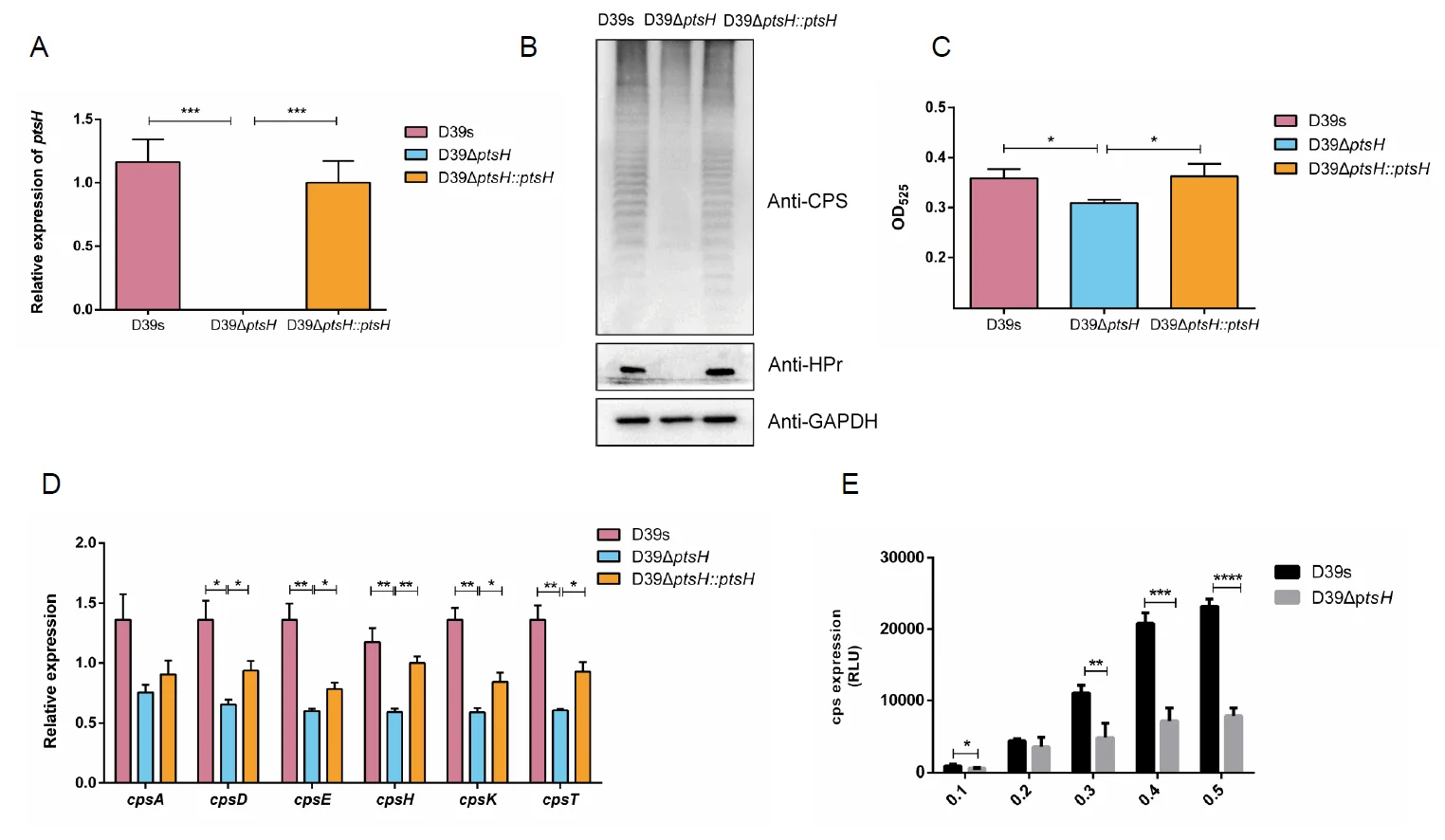
HPr regulates capsule synthesis through the *cps* locus. (A) Real-time fluorescence quantitative PCR to detect the transcript levels of *ptsH* in D39s, D39Δ*ptsH* and D39Δ*ptsH::ptsH*. (B) Western blot assay to determine the HPr protein expression level and CPS expression of D39s, D39Δ*ptsH* and D39Δ*ptsH::ptsH*. (C) Uronic acid assay to determine the CPS content in D39s, D39Δ*ptsH* and D39Δ*ptsH::ptsH*. (D) Real-time fluorescence quantitative PCR to detect the transcript levels of CPS synthesis-related genes in D39s, D39Δ*ptsH* and D39Δ*ptsH::ptsH*. (E) The luciferase reporter strains D39s-*Pcps-luc* and D39Δ*ptsH*-*Pcps-luc* were assayed for *cps* promoter activity. Data are shown as mean ± SEM of three independent experiments (n = 3). ^*^, p < 0.05; ^**^, p < 0.01; ^***^, p < 0.001; ns, not significant.

HPr is a synergistic effector of CcpA that affect CcpA activity by binding to the *cre* site (Fujita, 2009; Görke and Stülke, 2008). To investigate whether HPr has a regulatory effect on CPS synthesis, we constructed *ptsH* silent mutation (Δ*ptsH*) in the streptomycin-resistant D39-derived strain (D39s) by using the Janus cassette (JC) (Sung et al., 2001), the complemented mutant Δ*ptsH::ptsH* was generated by transforming the shuttle plasmid pIB166-*ptsH* into Δ*ptsH* mutant. Q-PCR and Western bolt analysis showed that the mRNA and protein were absent in Δ*ptsH* mutant and restored in Δ*ptsH::ptsH* (Fig. 4A and 4B). These results indicated that *ptsH* -deficient and *ptsH* -complementary strains were successfully constructed. Next, Western blot was performed to determine the production of CPS in D39s, Δ*ptsH*, and Δ*ptsH::ptsH* in normal C + Y medium. The result revealed that the content of CPS in Δ*ptsH* mutant was significantly reduced compared to the wild-type (Fig. 4B). To further confirm the western blot result, we quantified the amount of CPS of the strains by uronic acid assay. The uronic acid level of Δ*ptsH* was significantly lower than that of D39s, while the content was completely restored in the complemented strain Δ*ptsH::ptsH* (Fig. 4C), suggested that HPr promoted CPS synthesis.

CPS is a key virulence factor of *S. pneumoniae*. Mouse infection experiments were performed. We intranasally infected mice (pneumonia model) using 10^8^ CFU bacteria, and two days after infection, mice infected with D39s and Δ*ptsH::ptsH* began to die, with a nearly 50% reduction in survival, whereas almost all mice infected with Δ*ptsH* survived (Fig. S2A). In a low-dose pneumonia infection model (2 × 10^7^ CFU), we collected nasal lavage, blood, lung and spleen homogenates from mice in different infection groups 48 h after infection for plate counting, and the bacterial load in the Δ*ptsH* group was significantly lower than that in both the D39s and Δ*ptsH::ptsH* groups (Fig. S2B, S2C, S2D, and S2E). These results suggested that the deletion of *ptsH* resulted in a significant reduction in virulence.

Next, we examined the mRNA levels of *cps2A*, *cps2D*, *cps2E*, *cps2H*, *cps2K*, and *cps2T* in D39s, Δ*ptsH*, and Δ*ptsH::ptsH* strains. The mRNA levels of Δ*ptsH* were down-regulated relative to D39s, whereas were partially restored in the complemented strain Δ*ptsH::ptsH* (Fig. 4D). Additionally, we constructed the luciferase reporter strain D39Δ*ptsH*-*Pcps-luc* to observe the effect on *cps* promoter activity. The result showed that deletion of *ptsH* resulted in a significant reduction in promoter activity (Fig. 4E). These results suggested that HPr positively regulated CPS biosynthesis by influencing the transcription of the *cps* operon.

### HPr is involved in the regulation of glucose on capsule synthesis

**Fig. 5.**
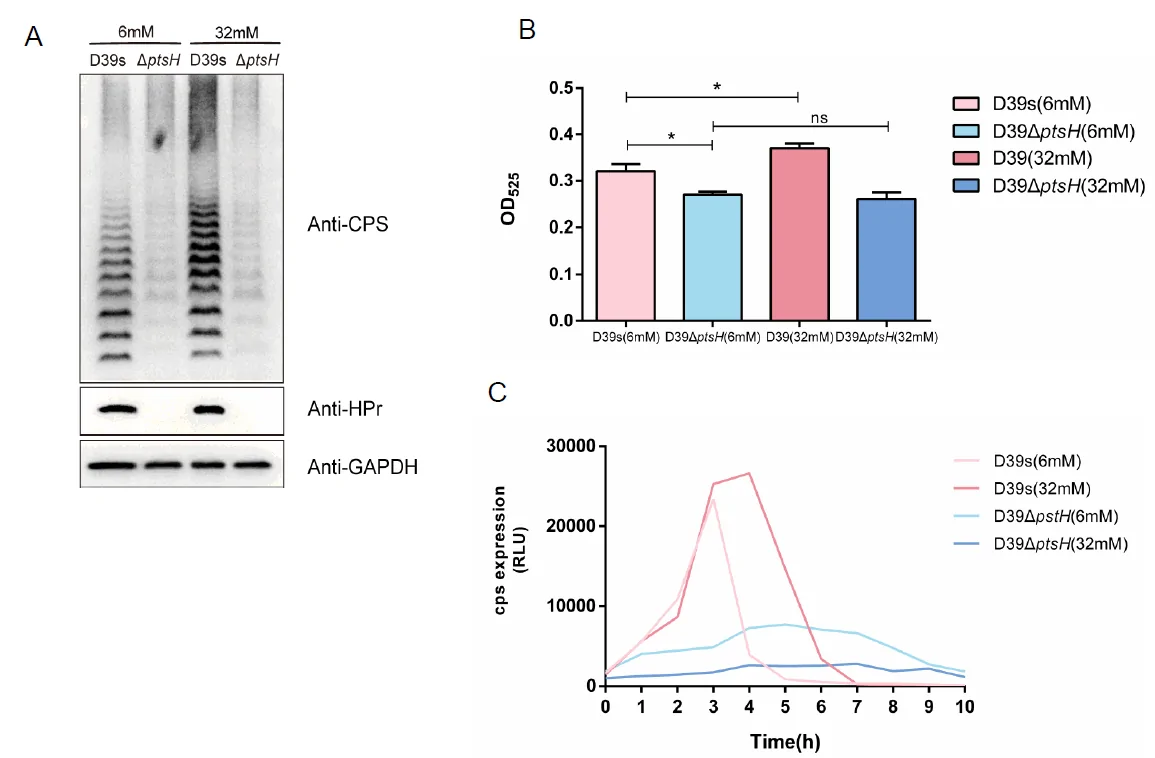
HPr is involved in the regulation of glucose on capsule synthesis. (A) Western blot assay to determine the CPS expression of D39s and D39Δ*ptsH* at different glucose concentrations. (B) Uronic acid assay to determine the CPS content of D39s and D39Δ*ptsH* at different glucose concentrations. (C) Luciferase reporter strains D39s-*Pcps-luc* and D39Δ*ptsH* *Pcps-luc* were assayed for *cps* promoter activity at different glucose concentrations. Data are shown as mean ± SEM of three independent experiments (n = 3). ^*^, p < 0.05; ^**^, p < 0.01; ^***^, p < 0.001; ns, not significant.

To investigate whether HPr is also involved in the regulation of CPS synthesis by glucose, we performed Western blot (Fig. 5A) and uronic acid assay (Fig. 5B) on D39s and Δ*ptsH* grown in C + Y medium supplemented with 6 mM and 32 mM glucose concentrations, which showed that, similar to Δ*ccpA*, there was almost no difference in the CPS content of Δ*ptsH* in different glucose concentrations. we also assessed the transcript levels of the *cps2A*, *cps2B*, *cps2C*, and *cps2D* genes (Fig. 3B), the expression of the genes was down-regulated and did not increase with higher glucose concentration in Δ*ptsH* compared to wild-type. the same trend was also shown in the activity assay of the *cps* promoter (Fig. 5C). These results suggested that HPr was involved in the glucose regulation of CPS.

### Glucose promotes the formation of HPr-Ser46~P to promoting the regulation of CcpA on the capsule

It has been proposed that in group A *streptococci* (GAS), HPr is able to sense extracellular glucose concentration to phosphorylate or dephosphorylate itself, then to bind or dissociate from CcpA, which leads to changes in the binding of CcpA to *cre* (Shelburne et al., 2008). Studies reported that in *B. subtilis*, CcpA specifically interacted with HPr-Ser46~P to play a regulatory role, whereas dephosphorylated HPr, HPr-His15~P, and bisphosphorylated [HPr(Ser46~P)(His15∼P)] did not bind to CcpA (Deutscher et al., 1995). Therefore, we hypothesized that in *S. pneumoniae*, glucose may also affect CcpA-mediated regulation by influencing HPr-Ser46~P.

**Fig. 6.**
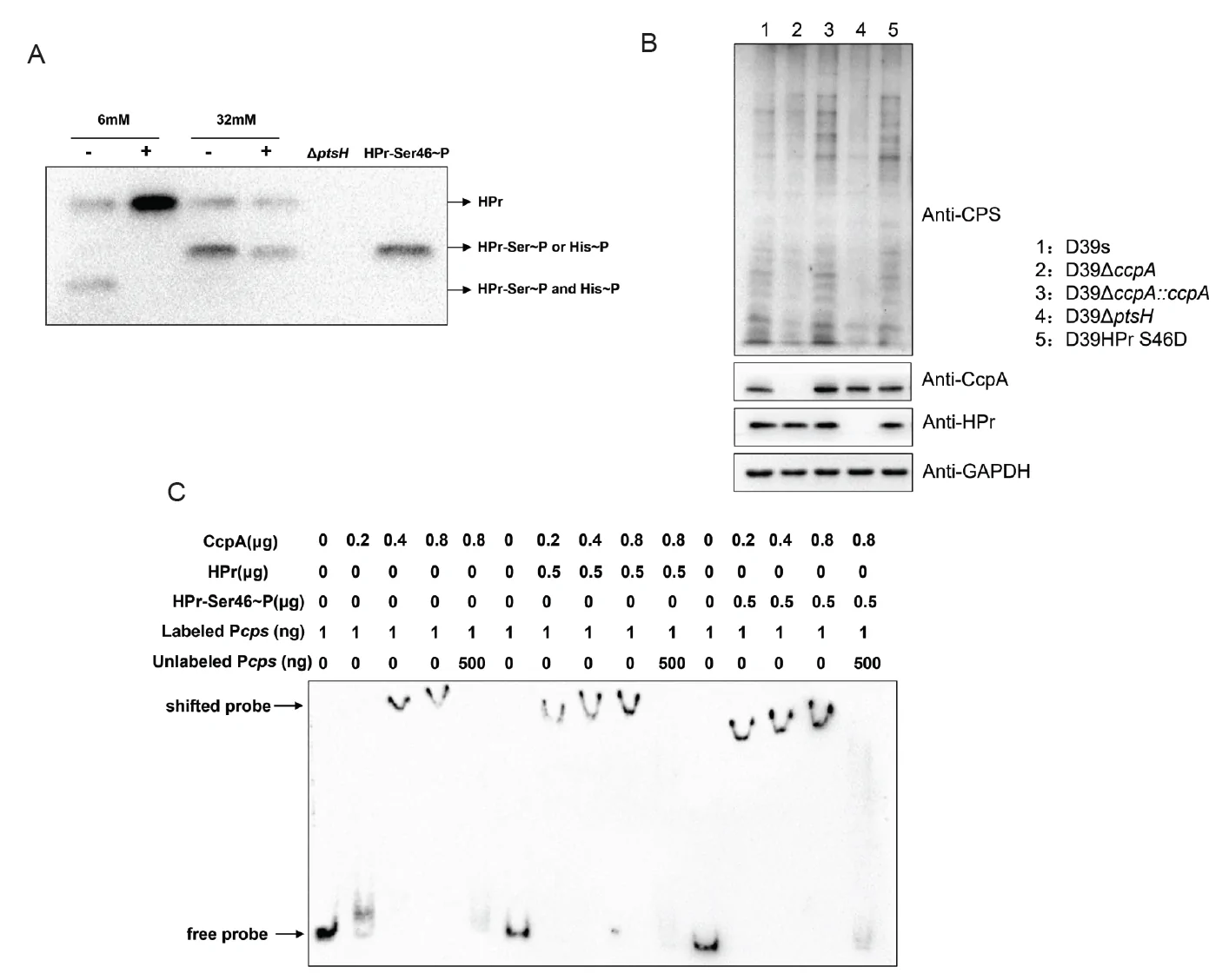
Glucose promotes the formation of HPr-Ser46~P to promoting the regulation of CcpA on the capsule. (A) Non-denaturing gel analysis of the HPr and its phosphorylated forms (HPr-His15~P or HPr-Ser46~P) to determine the amount of HPr-Ser46~P at different glucose concentrations in WT strains. The *ptsH* mutant as negative control, HPr S46D strain as positive control (Equal amounts of cell extracts untreated [−] or incubated at 70°C for 10 min [+] to hydrolyze the heat-labile HPr-His15~P). (B) Western blot assay to determine the expression of CPS in HPr S46D mutant bacteria at 6mM glucose concentrations. (C) EMSA to detect the effect of the addition of HPr and HPr-Ser46~P proteins on the binding of CcpA to *cps* promoter.

To verify the above conjecture, we performed immunoblotting with anti-*S. pneumoniae* HPr antibody to determine the phosphorylation status of HPr in wild-type strains grown at different glucose concentrations. Since the amino phosphate bond of HPr-His15~P is thermally unstable, whereas the phosphate bond of HPr-Ser46~P is stable (Roy et al., 2008), we distinguished HPr-His15~P and HPr-Ser46~P that migrated to the same location by boiling the samples. The result showed that wild-type strains growing at a low glucose concentration (6 mM) had distinct bands corresponding to the double phosphorylated form of HPr [HPr (Ser46~P)(His15~P)] and dephosphorylated HPr, whereas strains grown at high glucose concentration (32 mM) were predominantly monophosphorylated forms of HPr-His15~P and HPr-Ser46~P (Fig. 6A).

We further attempted to construct serine residue mutations of HPr, which are commonly used to study HPr-mediated CCR in Gram-positive bacteria: HPr S46A and HPr S46D, but HPr S46A, the HPr mimic dephosphorylation mutation, is lethal in *S. pneumoniae* (Fleming et al., 2015). we only successfully constructed HPr S46D simulated phosphorylation mutant and investigated its effect on CPS synthesis. Western blot result showed that the CPS content of the HPr S46D strain was significantly enhanced compared with D39s, indicating that increased phosphorylation of HPr-Ser46~P indeed promoted CPS synthesis (Fig. 6B).

Since the literature suggested that HPr-Ser46~P may affect the binding of CcpA to the promoters of target genes, we examined the ability of CcpA protein to bind to the *cps* promoter in the absence or presence of HPr and HPr-Ser46~P proteins (Fig. 6C). The result showed that both HPr and HPr-Ser46~P significantly enhanced the binding of CcpA to the *cps* promoter, with the latter having a higher enhancement, suggesting that HPr-Ser46~P can promote the binding of *cps* promoter to CcpA more strongly than the prototype.

Overall, the increase in glucose concentration promoted the formation of HPr-Ser46~P, which facilitated the binding of CcpA to the *cps* promoter. This coordinated interaction promoted the expression of *cps* genes and ultimately led to an increase in CPS production.

## Discussion

*Streptococcus pneumoniae* is a major pathogen causing pneumonia, otitis media, meningitis and sepsis globally, leading to over 500,000 childhood fatalities each year (Briles et al., 2019). CPS is the most crucial virulence factors. During pathogenesis, *S. pneumoniae* adapts to various host ecosystems by fine-tuning the transcript levels of its *cps* genes and controlling CPS production. Many carbon sources exist in the host ecological niche, such as the nasopharynx, which is predominantly rich in galactose, N-acetylglucosamine (GlcNAc) and mannose, or blood, which is dominated by glucose (Aprianto et al., 2018; Hobbs et al., 2018). Recent report indicates that various exogenous carbohydrates can affect CPS production and gene expression (Werren et al., 2023). For example, in glucose-enriched blood, the increased transcript levels of the *cps* gene promote CPS synthesis (Kim et al., 1999). This may be an important reason why diabetes is considered an independent risk factor for severe *S. pneumoniae* infection (Ishiguro, 2016). However, the specific factors involved in this regulation remain unclear.

CcpA is a major regulator of carbohydrate metabolism. Previous studies have shown that in *Streptococcus suis*, CcpA deficiency can reduce CPS synthesis (Willenborg et al., 2011), and Δ*ccpA* mutant strains of group A *streptococci* have significantly reduced virulence in invasive mouse models (Sonenshein, 2007). However, the specific mechanism of CcpA on CPS synthesis in *S. pneumoniae* serotype 2 strains D39 has not been reported. In this study, we elucidate the pivotal role of CcpA in the regulation of carbohydrate metabolism and CPS.

We discovered that CcpA promotes CPS synthesis in an environment where glucose is the sole carbon source, indicating that CcpA may act as a transcriptional regulator of the *cps* operons. To investigate this, we first analyzed the *cps* promoter sequence and identified a conserved CcpA binding motif. We then experimentally confirmed that CcpA specifically binds to the *cps* promoter, facilitating the transcription of *cps* loci and thereby promoting CPS synthesis. These findings demonstrated that CcpA positively regulated CPS synthesis in glucose as the sole carbon source.

We observed that glucose positively regulates CPS synthesis in wild-type strains, whereas the effect disappeared after *ccpA* deficiency, suggesting that CcpA may be involved in the regulation. Further findings revealed that high glucose promoted the transcriptional activity of the *cps* promoter, but this effect was eliminated after CcpA deletion. These evidences suggested that the regulatory effect of glucose should be realized through the transcriptional regulation of the *cps* locus by CcpA. Interestingly, we found that neither the level of CcpA protein expression nor its ability to bind to the *cps* promoter was affected by glucose concentration, which was consistent with the results reported in *Bacillus subtilis* (Mijakovic et al., 2005), implying that alternative regulatory mechanisms are likely involved in glucose regulation of CPS synthesis.

In many bacteria, CcpA functions as a regulatory protein by forming a complex with HPr-Ser46~P, enabling specific binding to *cre* sequences. In *Bacillus subtilis*, the interaction between CcpA and HPr-Ser46~P enables CcpA to specifically recognize *gnt* cis-acting catabolic response elements (Fujita et al., 1995). Additionally, the activation of *ilv-leu* catabolism metabolites also requires the presence of CcpA and the HPr-Ser46~P complex (Fujita et al., 2014). In *Streptococcus suis*, the protein HP0197 promotes the phosphorylation level of phosphocarrier protein HPr at residues Ser-46, which enhances the binding of CcpA to *cps* promoter and thus regulates carbohydrate utilization and CPS synthesis gene expression (Willenborg et al., 2011). Therefore, we speculated that HPr-Ser46~P regulated CPS synthesis by promoting the binding of CcpA to the *cps* promoter. This hypothesis was confirmed by EMSA experiments. Compared with unmodified HPr protein, HPr-Ser46~P protein significantly promoted the binding of CcpA protein to *cps* promoters. Meanwhile, it was also observed that the form of HPr-Ser46~P protein increased significantly with the increase of glucose concentration, suggesting that glucose promoted CPS synthesis by promoting the phosphorylation of HPr-Ser46.

Studies have shown that HPr has two phosphorylation sites in low G + C Gram-positive bacteria: His-15 site and Ser-46 site. It has been reported that the HPr-His15~P protein form of *Bacillus subtilis* cannot bind to CcpA (Reizer et al., 1996). But another study has shown that HPr-His15~P in *Bacillus subtilis* may exerts its regulatory effect by stimulating the binding of CcpA and *cps* promoter through FBP and Glc-6-P (Horstmann et al., 2007). Our results shown that the form of HPr-His15~P protein also increases significantly when glucose concentration increases (Fig. 6A), suggesting that glucose may also promote capsule synthesis by promoting the phosphorylation of His15. Further research is needed to determine whether it has the same regulatory mechanism as *Bacillus subtilis*.

Based on the existing experimental evidence, we elucidated the hypothesis that glucose regulates CPS synthesis via CcpA in the presence of glucose. It promotes the formation of HPr-Ser46~P, which synergistically interacts with CcpA to promote CPS synthesis by facilitating the binding of CcpA to the *cps* promoter. These findings in this study reveal a novel mechanism for the regulation of CPS synthesis by carbon metabolism, which provides a new target for the prevention and treatment of *S. pneumoniae*.

## Supplementary Information

Table S1. Bacterial strains and plasmids used in this study

Table S2. Primers used in this study

Fig. S1. Overproduction and purification of CcpA, HPr and HPr-Ser46~P. SDS-PAGE to detect the expression and purification of CcpA protein.

Fig. S2. Deletion of *ptsH* reduces virulence. (A) Survival of mice pneumonia model infected with D39s, D39Δ*ptsH*, and D39Δ*ptsH::ptsH* (bacterial load of 10^8^ CFU) (n = 10). (B, C, D, and E) Nasal lavage, lung homogenate, spleen homogenate, and cardiac blood bacterial load in mice infected with different *S.pn* strains D39s, D39Δ*ptsH*, and D39Δ*ptsH::ptsH* (bacterial load of 2 × 10^7^ CFU) for 48h (n = 5). Survival was analyzed using log-rank tests. ^***^, P < 0.001.
