# Supplementary material for: Glucose affects capsular polysaccharides synthesis via CcpA and HPr in Streptococcus pneumoniae
Source: J Microbiol. 2025 May 27;63(5):e2411024. doi: 10.71150/jm.2411024 (PMC13577091; doi:10.71150/jm.2411024)

Fig. S1. Overproduction and purification of CcpA, HPr and HPr-Ser46~P. SDS-PAGE to detect the expression and purification of CcpA protein.

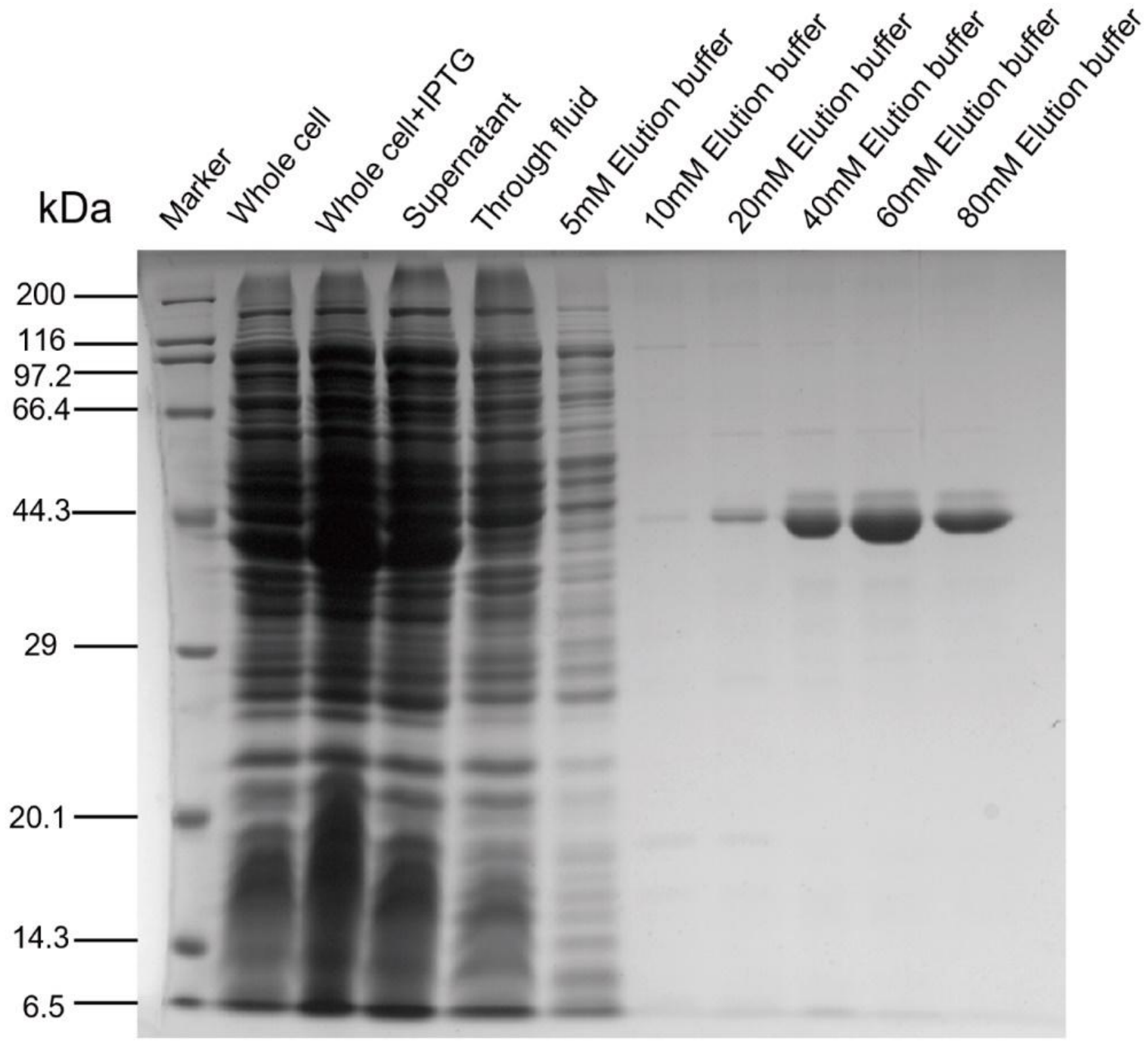

Supplement: Supplementary file 1 [file jm-2411024-Supplementary-Fig-S1.pdf]
