# Supplementary material for: Glucose affects capsular polysaccharides synthesis via CcpA and HPr in Streptococcus pneumoniae
Source: J Microbiol. 2025 May 27;63(5):e2411024. doi: 10.71150/jm.2411024 (PMC13577091; doi:10.71150/jm.2411024)

Fig. S2. Deletion of *ptsH* reduces virulence. (A) Survival of mice pneumonia model infected with D39s, D39Δ*ptsH*, and D39Δ*ptsH::ptsH* (bacterial load of 10^8 CFU) (n = 10). (B, C, D, and E) Nasal lavage, lung homogenate, spleen homogenate, and cardiac blood bacterial load in mice infected with different *S.pn* strains D39s, D39Δ*ptsH*, and D39Δ*ptsH::ptsH* (bacterial load of 2×10^7 CFU) for 48h (n = 5). Survival was analyzed using log-rank tests. ***, P < 0.001.

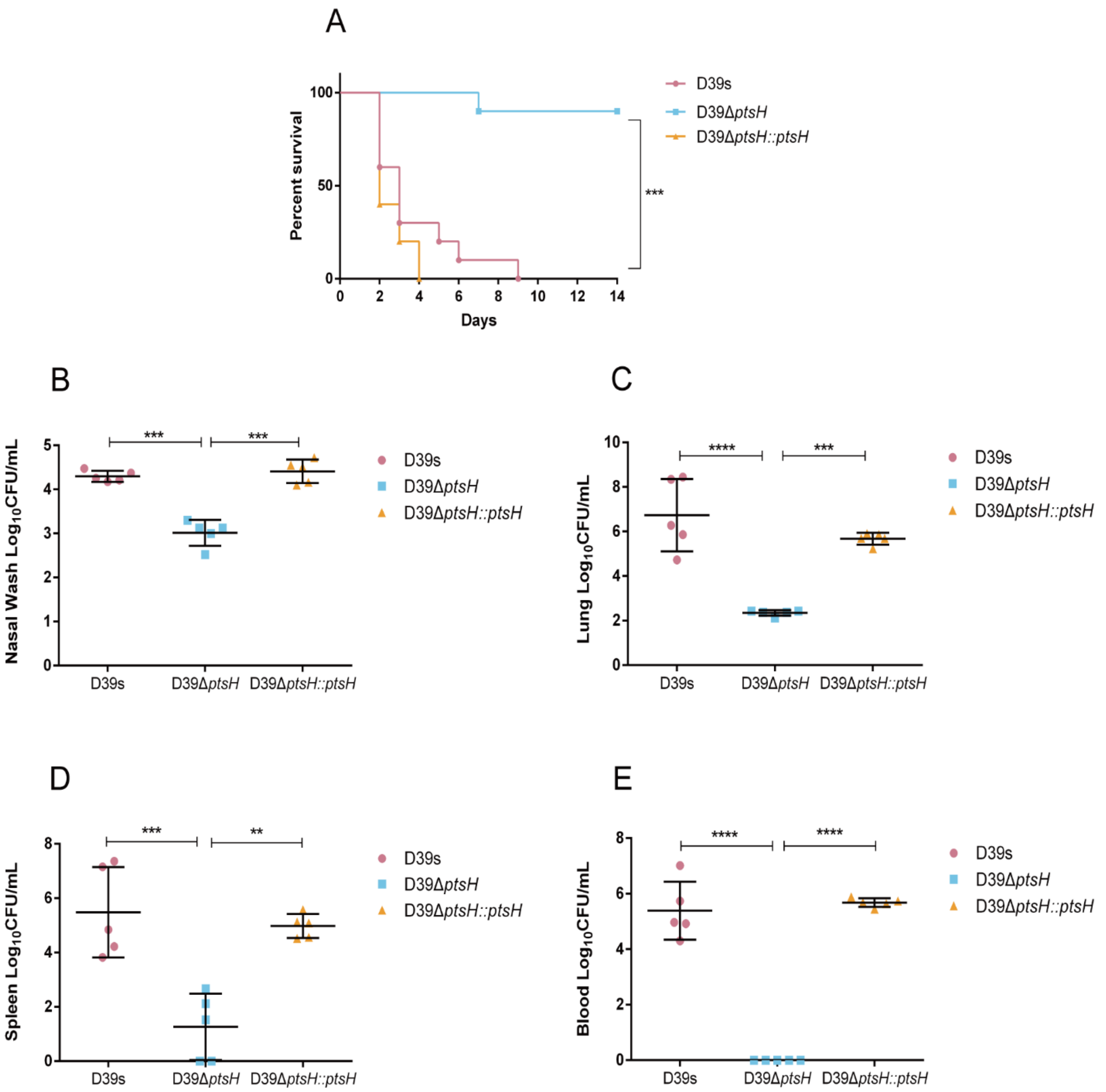

Supplement: Supplementary file 2 [file jm-2411024-Supplementary-Fig-S2.pdf]
