# Supplementary material for: Glucose affects capsular polysaccharides synthesis via CcpA and HPr in Streptococcus pneumoniae
Source: J Microbiol. 2025 May 27;63(5):e2411024. doi: 10.71150/jm.2411024 (PMC13577091; doi:10.71150/jm.2411024)
Supplement: Supplementary file 3 [file jm-2411024-Supplementary-Table-S1.pdf]

Table S1. Bacterial strains and plasmids used in this study

Strain or plasmid	Description	Antibiotic resistance	Reference or source
**Strains**			
D39s	D39 derivative, rpsL1	Sm^R	This study
D39Δ*ccpA*	Complete replacement of *ccpA*	Sm^R	This study
D39Δ*ccpA::ccpA*	Ectopic expression of *ccpA*	Chl^R	This study
D39Δ*ptsH::JC*	Replacement of *ptsH* by Janus cassette	Sm^R	This study
D39Δ*ptsH*	Complete replacement of *ptsH*	$Kana^R$	This study
D39Δ*ptsH::ptsH*	Ectopic expression of *ptsH*	Chl^R	This study
HPr S46D	Phosphorylation at serine 46 of *ptsH*	Sm^R	This study
D39s-*Pcps-luc*	P*cps* luciferase reporter plasmid was inserted into D39s	Chl^R	This study
D39Δ*ccpA-Pcps-luc*	P*cps* luciferase reporter plasmid was inserted into D39Δ*ccpA*	Chl^R	This study
D39Δ*ptsH-Pcps-luc*	P*cps* luciferase reporter plasmid was inserted into D39Δ*ptsH*	Chl^R	This study
**Plasmids**			
pIB166-*ccpA*	Shuttle vector carrying *ccpA*	Chl^R	This study
pIB166-*ptsH*	Shuttle vector carrying *ptsH*	$Kana^R$	This study
pET-28a-CcpA	Plasmid expressing CcpA protein	$Kana^R$	This study
pET-28a-HPr	Plasmid expressing HPr protein	$Kana^R$	This study
pET-28a-HPr-Ser46~P	Plasmid expressing HPr-Ser46~P protein	$Kana^R$	This study
