# Supplementary material for: Glucose affects capsular polysaccharides synthesis via CcpA and HPr in Streptococcus pneumoniae
Source: J Microbiol. 2025 May 27;63(5):e2411024. doi: 10.71150/jm.2411024 (PMC13577091; doi:10.71150/jm.2411024)
Supplement: Supplementary file 4 [file jm-2411024-Supplementary-Table-S2.pdf]

Table S2. Primers used in this study

Primer		Sequence (5´-3´)
**Primer for Construction of strains**		
ΔptsH	Pr1440	GATTTGTGTGCGGAACATAG
	Pr1441	TTTCAGCATTATCCTCTAGAGGGAAATGACAGAAATGCTT
	Pr1442	AGCATAAGGAAAGGCTCGAGAGTTTGACTCTCCTTTTGTT
	Pr1443	AGGAGTAGCAGGTTGGTAGC
	YR0013	AACAAAAGGAGAGTCAAACTGGGAAATGACAGAAATGCTT
	YR0014	AAGCATTTCTGTCATTTCCCAGTTTGACTCTCCTTTTGTT
ΔptsH::ptsH	Pr1963	TGTTCATGAAATTAAGGAACGAA
	Pr1964	CCATGACAGCCATGATAATTACT
	YR0019	CGGGATCCATGGCTTCTAAAGATTTCCA
	YR0020	CCGCTCGAGATTTTTGGTCATTAATGGTGATGGTGATGGTGTGCCAAT CCTTCTTTTTCCA
**Primer for the expression of recombinant protein**		
HPr	Pr1419	CCCATATGATGGCTTCTAAAGATTTCCA
	Pr1420	CCCTCGAGTTATGCCAATCCTTCTTTTT
HPr-Ser46~P	Pr1431	GAAGATCTTCTTAAGGAGGCAAATATGGCTTCTAAAGATTTCCA
	Pr1432	CATAACACCCATAATGTCTTTAAGGTTAACTGA
	Pr1433	TCAGTTAACCTTAAAGACATTATGGGTGTTATG
**Primer for qPCR**		
gyrB	gyrB F	GTTCGTATGCGTCCAGGGAT
	gyrB R	ATACCACGCCCATCATCCAC
ccpA	Pr1322	ATGAGTTGGCAGCAGGAGT
	Pr1323	GACGGGTAAAGCGTGAGAT
ptsH	Pr1601	GCCAACACCAAGACTCATAACAC
	Pr1602	CACGTAGTGGCAGAAACAGG
cps2A	cps2A F	TTGTCAGCTCTGTGTCGCTC
	cps2A R	TTATCAGTCCCAGTCGGTGC

cps2B	cps2B F	CTACCTCTCACCGTCGCAAG
	cps2B R	CAGCCCCGTAAGCAATGACT
cps2C	cps2C F	AAACAGCCAGAGGAAGCCAG
	cps2C R	GAAGGAGTCGTAGCTGGTCG
cps2D	cps2D F	TCCTGTCGGTGTCGTGATTG
	cps2D R	AAACGGCTTCCCTGTGTGTT
cps2E	cps2E F	AAGTGGTATTGGAAGCGGGC
	cps2E R	GGCTACCAACTCCCCAACAA
cps2F	cps2F F	TTAGTTGCTGCTAGCTTTGGGA
	cps2F R	TATTGACTTGTGTCGCCCCC
cps2G	cps2G F	ACAGCTTGGCAAACAGCCTA
	cps2G R	GCCTCCACCTCTTCCTTTCC
cps2H	cps2H F	TGGACTGGCTGATGGTTCTCT
	cps2H R	GACACCACCAAATCCTGACCC
cps2I	cps2I F	GGGAATGTTGTCTGGAGCGT
	cps2I R	TGTCCTCTCAAAGCAATTTCCA
cps2J	cps2J F	TTTTGTGGTGTAGCGGTGAA
	cps2J R	ATTCCACCAGCAAATCCTGA
cps2K	cps2K F	CCTTCCTTCGGCTATGGTGG
	cps2K R	ACGTGTCGAATTGCTCCGTA
cps2L	cps2L F	CGGTCCAGGTTTGAGCAAGA
	cps2L R	GCATAGTTGGAGCGAGGACA
cps2M	cps2M F	CGTGGCTGGTTTAAGGAAAA
	cps2M R	TCAACCCAAGAACCCAGAAC
cps2N	cps2N F	CCAAGCTCGCCTTACTCATC
	cps2N R	AGTTGGAAATCGTTGCCTTG
cps2O	cps2O F	GTTGATGCAGCAGAGGATGA
	cps2O R	CCCACTCTTGTCCAACTGGT
cps2P	cps2P F	TGAGAAGCAATGGGAACGCT

	cps2P R	TCCACTCCTAGCCCAACTTC
cps2T	cps2T F	AGTGGGAGGGACCAATCCAT
	cps2T R	GGCACCATTCTCTCCGACTT
**Primer for EMSA**		
	P*cps* F	TACACATCTGCTTCTAAAATATTGT
	P*cps* R	TTAAAACGTCTACTCATGATTAACA
	P*cps*-bio R	TTAAAACGTCTACTCATGATTAACA（5‘biotin）
